# AI-Based Forecasting of Polymer Properties for High-Temperature Butyl Acrylate Polymerizations

**DOI:** 10.1021/acspolymersau.4c00047

**Published:** 2024-07-26

**Authors:** Jelena Fiosina, Philipp Sievers, Marco Drache, Sabine Beuermann

**Affiliations:** † Institute of Informatics, Clausthal University of Technology, Julius-Albert-Str. 4, 38678 Clausthal-Zellerfeld, Germany; ‡ Institute of Technical Chemistry, Clausthal University of Technology, Arnold-Sommerfeld-Str. 4, 38678 Clausthal-Zellerfeld, Germany

**Keywords:** polymer microstructure, machine learning, kinetic Monte Carlo simulation, regression, explainable AI

## Abstract

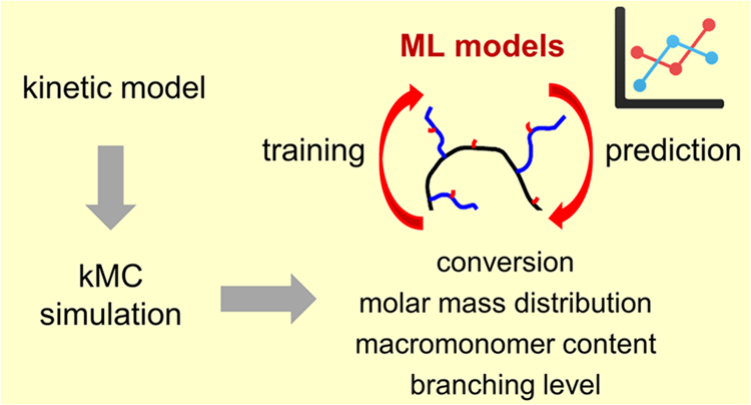

High-temperature polymerizations involving self-initiation of the monomer are attractive because of high reaction rate, comparable lower viscosities, and no need for an additional initiator. However, the polymers obtained show a more complex microstructure, e.g., with specific branching levels or significant amounts of macromonomer. Simulations of the polymerization processes are powerful tools to gain a deeper understanding of the processes and the elemental reactions at the molecular level. However, simulations can be computationally demanding, requiring significant time and memory resources. Therefore, this study aims at applying AI-based forecasting of tailored polymer properties and using a kinetic Monte Carlo simulator for the generation of training and test data. The applied machine learning (ML) models (random forest and kernel density (KD) regression) predict monomer concentration, macromonomer content, and full molar mass distributions as a function of time, as well as the average branching level with an excellent performance (*R*^2^ (coefficient of determination) > 0.99, MAE (mean absolute error) < 1% for kernel density regression). This study explores the number of training data needed for reliable and accurate predictions in ML models. Explainability methods reveal that the importance of input variables in ML models aligns with expert knowledge.

## 1 Introduction

Acrylates represent a very important group of monomers for radical polymerization. Frequently, they are used for coatings, adhesives, and biomedical applications.^1^ Performing radical polymerizations at higher temperatures is attractive for technical applications because of high reaction rates, lower viscosities, and the access to polymers with lower molar masses.^2^ However, under these conditions, elemental reactions start to play a role that are negligible at lower temperatures.^2,3^ Examples are the self-initiation of monomer and β scission of the macroradical, the latter reaction resulting in the formation of macromonomers and its elemental reactions.^4−8^ Because of the self-initiation process, the initiation kinetics is very sensitive to changes in the monomer concentration.^9,10^

**Figure 1:**
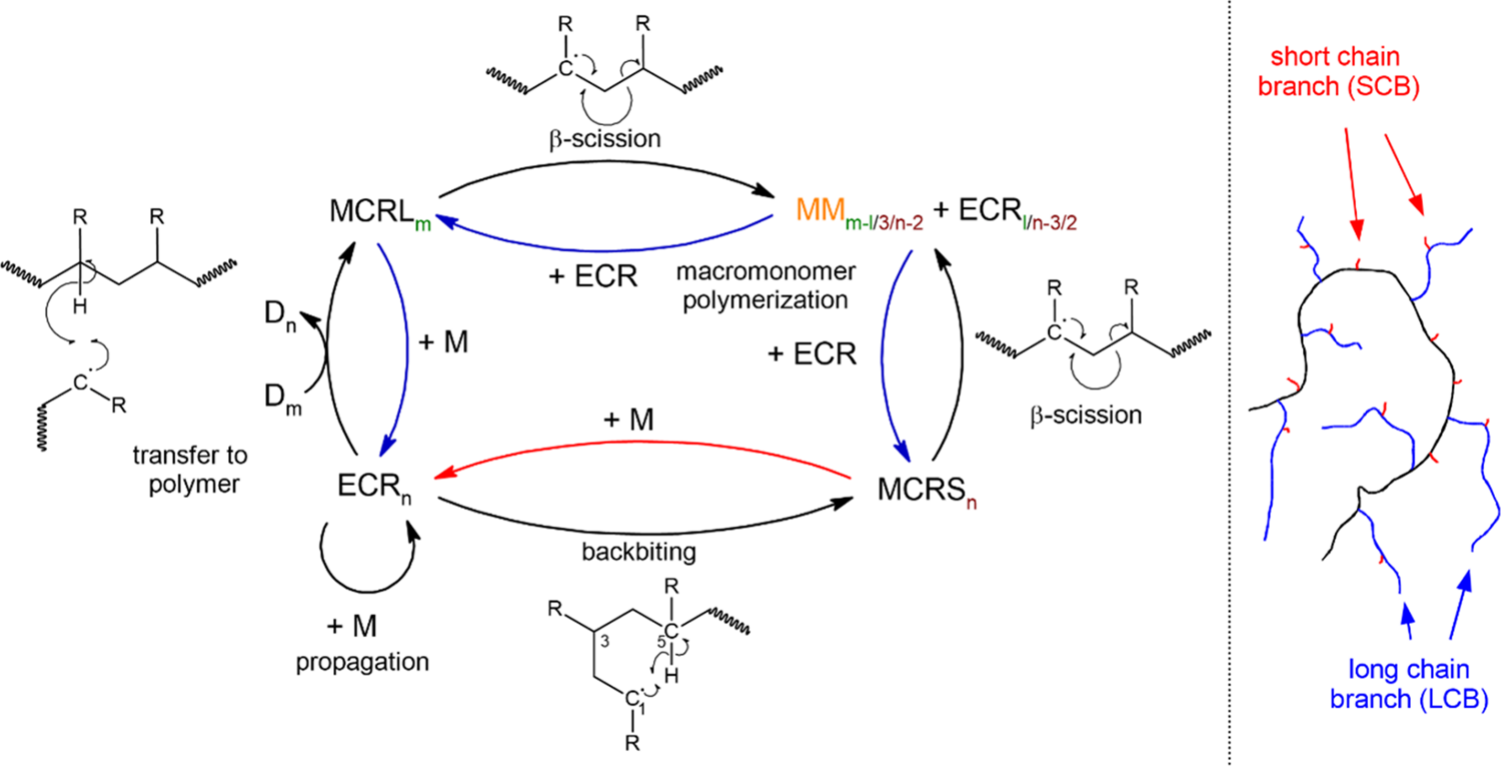
Cutout of the reaction mechanism leading to long chain branches (LCB, indicated by blue arrows on the right) and short chain branches (SCB, indicated by red arrows on the right). SCBs are formed from midchain radicals (MCRs) generated by backbiting of end-chain radicals (ECR). LCBs originate from transfer to polymer resulting in midchain radicals (MCRL), followed by the addition of a monomer or a macromonomer (MM). Macromonomers are formed by a β scission reaction of a midchain radical.

β scission occurs for midchain radicals (MCRs) that are either formed by backbiting or chain transfer to polymer reaction.^11^ In addition to decreasing the average molar mass, the occurrence of chain transfer followed by β scission leads to a distinct fraction of low molar mass species in the molar mass distribution (MMD).^12^ As indicated in Figure 1, β scission always results in a radical and a macromonomer species. Macromonomers undergo propagation and are built into a growing polymer chain,^13,14^ thus, leading to long chain branches (LCB). As shown in Figure 1, LCBs constitute long side chains of the polymer molecule. Much more frequent are small chain branches (SCBs), which are generated by a backbiting reaction, an intramolecular transfer that is favored due to its six-membered transition state. Therefore, the length of the resulting side chain is well-defined with a length of 4 carbon atoms. For a detailed description of the entire polymerization mechanism, the reader is referred to Ballard and Asua.^1^

The occurrence of the elemental reactions mentioned is reflected in the microstructure of the polymer molecules and consequently, in the properties of the polymers.^1^ For example, LCBs are well-known to affect the viscoelastic behavior of the polymer product.^15^ Due to the strong correlation between polymerization and the final polymer properties, reliable tools are required for the design of polymerization processes for tailored polymers.

A lab-based approach to this challenge is laborious, and in general, details on the polymer microstructure are determined offline after the end of the polymerization. Frequently, information on complex structural motifs, in particular, as a function of reaction time, is scarce or not available. This gap can be closed by simulation techniques such as differential equation solvers^16^ or kinetic Monte Carlo (kMC) simulations.^17,18^ Simulations provide more detailed information because they allow for monitoring the features of interest such as the polymer microstructure or the concentrations of all components throughout the reaction. Due to its discrete nature, kinetic Monte Carlo (kMC) methods allow for tracking the structure of each individual polymer chain within the simulation.^19^ The downsides of this approach are long simulation times and a high memory demand rendering kMC simulations computationally expensive. This is especially true for high-temperature acrylate polymerizations. Due to the complex microstructure of the resulting polymers the memory demand increases and can easily reach 100 GiB and more.^20^ Therefore, it is favorable to combine kMC simulations with AI-based forecasting techniques that are much faster after they are trained.

AI offers exciting possibilities for advancements in polymer research. Machine learning (ML) techniques like deep learning and ensemble learning (random forest,^21^ XGBoost,^22^ CatBoost^23^) can be powerful tools for modeling and optimizing polymerization processes.^24−27^ Key properties like conversion and molar mass distributions were successfully predicted, even with limited data sets.^27−31^ However, a major hurdle remains: the difficulty in acquiring a sufficient number of data for robust AI models. Due to very large variability of the products, polymer research often faces limitations compared to other fields in generating large data sets. This data scarcity poses a significant challenge, particularly for studying complex radical polymerizations.

Despite this obstacle, recent research demonstrates the potential of AI even with limited data. For instance, high-performance ML methods were successfully constructed for predicting properties of poly(butyl acrylate) (PBA) from radical polymerization at 60–80 °C using a relatively small data set.^32^ The random forest method proved particularly reliable in predicting complex properties like the molar mass distribution (MMD) when other methods struggled.^32^ Thus, the application of ML to even more intricate polymer architectures is promising.

The aim of this study is to find ML methods that can predict more complex MMD shapes as well as the polymer branching level. Kernel density (KD) regression^33^ is another ML method, which shows a high performance for small data sets with a low number of input variables. KD regression is a nonparametric ML method and uses kernel smoothing and probability density mechanisms to predict the variable of interest. It is very attractive if the aim is to obtain high-performance models with a limited number of data.^34^ The downside of this method is relatively high computation time; however, it is considerably smaller than for kMC simulations.

If ML methods are not “glass box” models, such as linear regression or decision trees, it is crucial to identify the explanations of the feature importance.^35^ Powerful ML methods like KD regression and ensemble learning (random forest) can be incredibly effective, but they often result in “black box” models.^36,37^ This lack of transparency can be problematic for researchers and users, who need to understand the model’s reasoning and trust its predictions. To bridge this gap, it is crucial to develop methods that explain how input features influence the model’s outputs. For this purpose, model-specific explainability methods are available, such as “mean decrease in impurity”^38^ for random forest or DeepLIFT^39,40^ for neural networks. Additionally, there are model-agnostic explainability methods, e.g., such as permutation importance,^35^ Shapley values,^41^ which can be applied to explain each type of ML models. For the prediction of polymerization properties, it was shown that various explainability methods lead to very similar results.^32^

## 2 Considered Process and Polymer Properties

The goal is to create a suite of ML models for each of the process and polymer properties of interest. The ML models were trained with data obtained from the kMC simulations. For clarity of presentation, throughout the text, “simulation” will refer to the kMC procedure and “modeling” will refer to the ML approach. Butyl acrylate (BA) was used as a monomer in radical polymerizations due to its large industrial relevance. The initiator was not required because of the self-initiation of the monomer as a consequence of the high temperatures applied.

### 2.1 Monomer Concentration and Macromonomer Content (MMC)

The effect of the initial monomer concentration (*c*_BA,0_) and temperature (*T*) on the time-dependent concentration of butyl acrylate (*c*_BA_(*t*)) was investigated.

The macromonomer content (MMC) is defined as the number of macromolecules *n*_MM_ divided by the mass of polymer *m*_p_
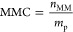
1

### 2.2 Molar Mass Distributions

The MMD describes the relation between the mass fraction *w*(log(*M*)) and the corresponding molar mass log(*M*). In the simulations, the kMC simulator generates the polymer MMD based on the distribution of chain lengths. This allows for determining the MMD for each time point during the polymerization process, considering factors like the chosen recipe and the reaction temperature. The MMD is always calculated for a constant number of 100 log(*M*) values.^32^ The corresponding *w*(log(*M*)) values are given as a vector, which represents the input data for the ML models.

**Figure 2:**
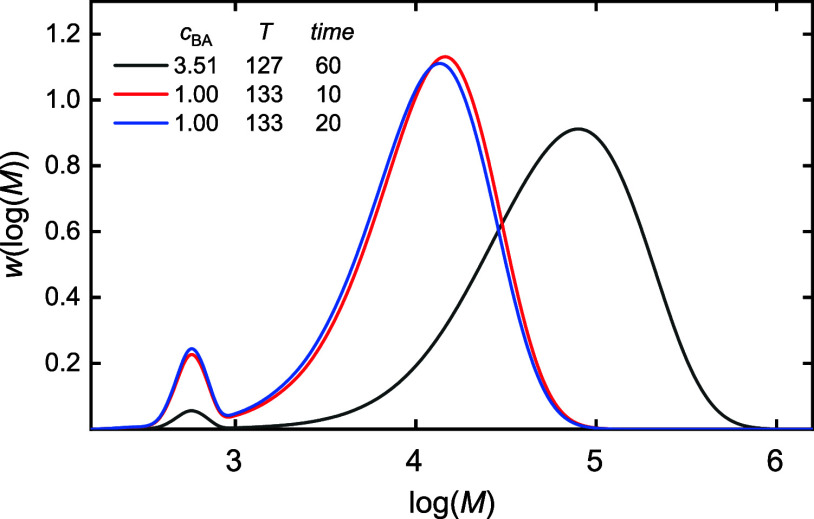
Example MMD results from kMC simulations for three different starting conditions, as indicated in the diagram. The units of *c*_BA_, *T*, and time are mol·L^–1^, °C, and min, respectively.

Figure 2 illustrates three different MMDs for different experimental settings. Contrary to MMDs related to low-temperature BA polymerizations, the distributions are characterized by two distinct peaks. The first peak at low molar masses is assigned to polymer chains, which were generated by a backbiting process, followed by a β scission reaction.

### 2.3 Average Branching Level

The average branching level (BL) of a chain length (*i*) is defined as the average number of LCB per polymer chain for all polymer chains with the given length *i*
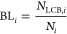
2

**Figure 3:**
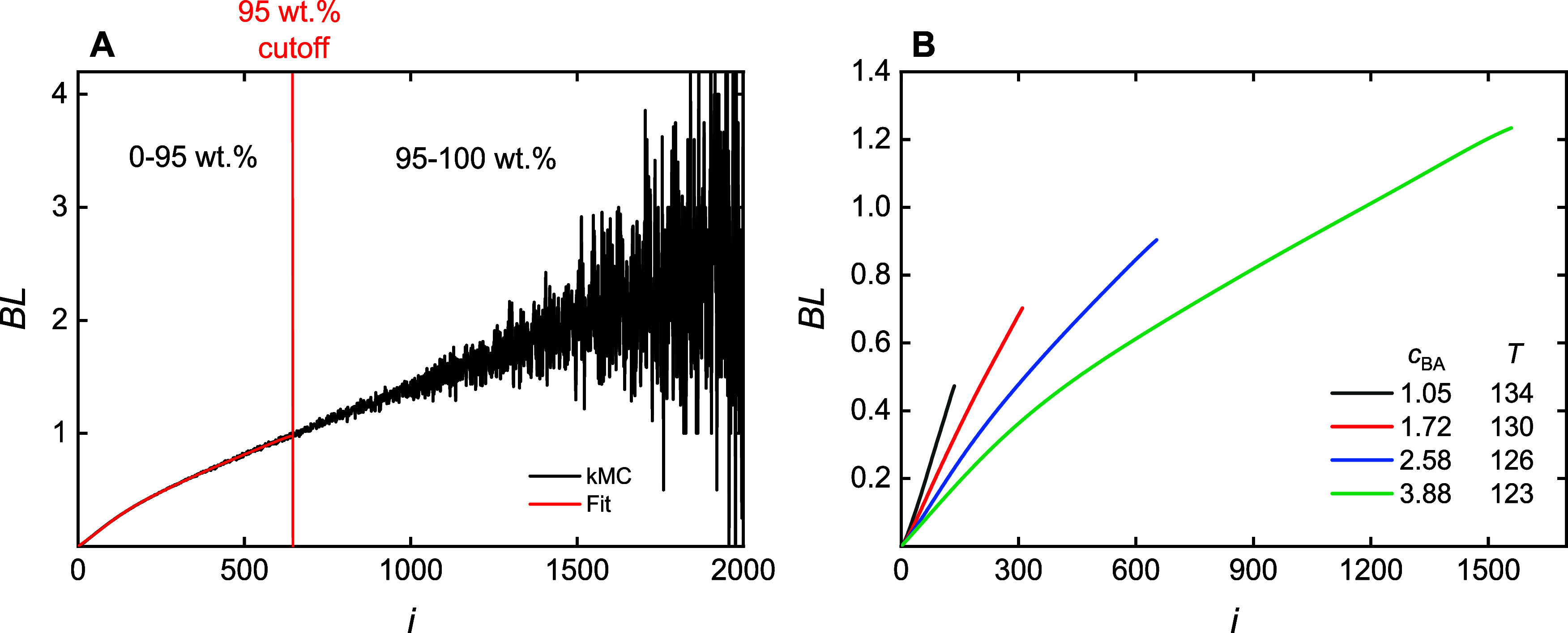
(A) example (2.75 mol·L^–1^, 130 °C) for data preparation: the red line indicates the 95 wt % threshold. Only data points on the left side of the threshold were used. A fit with a polynomial of degree 7 was used to further smoothen the data. (B) Four examples for the branching level after the processing described in (A). The units of *c*_BA_ and *T* are mol·L^–1^ and °C, respectively.

Determination of BL on the basis of data from kMC simulations poses a problem because kMC simulations are a discrete method. Therefore, each chain length is related to a distinct number of polymer molecules. At very high molar masses, the number of polymer molecules with a given high molar mass is either 0 or 1, and the data become very noisy in this molar mass range. Thus, as shown for the example in Figure 3A, only the lower 95 wt % of the polymer chains were used to train the ML models. The cutoff chain length or, in other words, the cutoff degree of polymerization is given by DP_95_ in the remainder. The BL data was further smoothed by fitting a polynomial of degree 7.

Figure 3B gives the branching levels of the four different experimental settings. The end points of the lines represent DP_95_. It is illustrated that different polymerization conditions are associated with very different values of DP_95_. The chain lengths for a given example are scaled and normalized with respect to this limiting value, which is necessary for building a general ML model for the BL prediction.

## 3 Methods

### 3.1 Considered ML Methods and Their Explainability

The properties and the associated equations described above can be predicted with the help of data-driven ML methods. This prediction task is represented as a multivariate regression problem,^42^ in which the input variables are initial monomer concentration *c*_BA,0_, temperature *T*, as well as time *t*, and the output variables *Y*(*t*) corresponding to the property of interest.
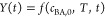
3

In this study, two ML methods were applied. The random forest method^21^ is the most popular ensemble method, which showed very good performance and stability, especially for multioutput regression in our previous research.^32^ RF regression leverages the power of multiple decision trees. Each tree, like a decision flowchart, splits data based on features to predict a continuous outcome. This ensemble approach strengthens predictions and prevents overfitting by averaging forecasts from diverse trees. The diversity comes from the use of random subsets of data and features to build each tree. Moreover, the variables’ importance in this method can be accessed with a model-specific feature explainability method ”mean decrease in impurity”.^38^ In contrast, decision trees, the building blocks of a RF, are inherently easy to understand and can handle both linear and nonlinear relationships within the data.

The second ML method applied is a kernel density regression.^33^ This is a nonparametric technique, which comes from statistics using kernel smoothing and probability density mechanisms to predict the variable of interest. KD regression estimates the probability distribution of a continuous outcome variable (*Y*) for a given input variable (*X*). Unlike traditional regression models that force the data to fit a particular shape (such as a line or curve), KD regression adopts a data-driven approach. For data points scattered on a graph, the KD regression builds a smooth curve by considering each data point and its neighbors. It uses a predefined function (called a kernel) to assign importance to these neighbors. Points closer to the point of interest have a greater influence on the final curve. By summing these influences from nearby data points, KD regression estimates the likelihood of encountering a particular outcome value for a given input value. This flexible approach allows KD regression to capture complex and potentially nonlinear relationships between the input and output variables. Compared to RF regression, KD regression does not support multitarget regression (MTR) by default, and an ensemble of single-target regression models should be considered, where each model separately predicts the dependent variable for an output.^43^ The method provides high performance; however, it is computationally very demanding. Thus, its application is reasonable in the case of small data set sizes. Here, KD regression with Gaussian kernels is applied. KD regression uses bandwidths as smoothing parameters, which depend on the data dimensionality and the scales of variables. Both affect the performance of an estimator. “Rule-of-thumb” method bandwidth estimators were used, such as Silverman’s rule^44^ or Scott’s rule^45^ combined with the Akaike’s Information Criterion (AIC) bandwidth estimation method.^46^ Since KD regression is a “black-box” model, in this study, a model-agnostic explainability method “permutation importance”^35^ was applied to evaluate feature importance. The method was selected, because of its simplicity and previous experience,^32^ where the model explainability results were similar compared to “Shapley values”, another popular explainability method.^41^ Thus, in this study, only one explainability method was selected for each ML model: a built-in “mean decrease in impurity”^38^ method for RF and a model-agnostic method “permutation importance” for nonparametric KD regression were used.

Different ML models employ various methods to interpret the influence of input features, which pose a challenge for the comparison of raw “permutation importance” scores measuring feature impact between models. Permutation importance is typically unscaled, meaning that its values do not necessarily sum up to a specific value. To enable a fair comparison of explainability across models, particularly when one model is a normalized decision-tree-based ensemble like a random forest, a scaling step was applied to the permutation importance results. The scaling ensures that the sum of all feature contributions equals 1, allowing for a more direct comparison of feature importance across different models.

### 3.2 Data Acquisition by kMC Simulation

kMC simulations, pioneered by Gillespie^47^ and further developed for complex systems,^18^ constitute a powerful technique, which offers detailed insights into the microstructure of individual polymer molecules.^19,48−55^ In this study, the in-house developed, open-source kMC simulator is applied to generate data for training ML models.^19^ mcPolymer has a proven track record in modeling various polymerization processes, including reversible deactivation radical polymerizations,^19,56^ acrylate polymerizations,^57^ and self-initiated butyl acrylate polymerizations.^20^ Previously, the kMC simulator was successfully used for generating training data for ML methods^32^ and for solving a reverse engineering problem by multiobjective optimization.^58^

While kMC simulations generate highly detailed information on polymer microstructure, the raw data are not directly usable for training ML models. To bridge this gap, a data pipeline was implemented to transform the kMC output into a format suited for input into ML models. This pipeline involves several steps. First, raw data are filtered to extract relevant features and undergo abstraction steps (e.g., calculation of an MMD). Then, the data is preprocessed for consistency and further organized into a well-structured format. Finally, the processed data are stored in MongoDB, a NoSQL database known for its flexibility and scalability.^59^ This user-friendly interface enables efficient storage and retrieval of training, test, and validation data for various ML models and the corresponding polymerization products.

## 4 Results and Discussion

### 4.1 Generation of Training Data and Experimental Design of ML Models

kMC simulations were employed to generate training and test data for the ML models. The kinetic model accounting for all elemental reactions is well established and was reported previously.^1,11,20^ The simulations were performed for radical polymerizations of butyl acrylate in the solvent 2-octanone for temperatures ranging from 120 to 140 °C and initial BA concentrations of 0.5 to 5.0 mol·L^–1^. A uniformly distributed grid approach resulted in 273 simulations covering this parameter space. Each polymerization process was simulated for a constant time of 60 min, with BA concentration and *MMC* recorded every 5 min and for other properties of interest recorded every 10 min. This yields 12 data points for BA concentration and MMC as well as 6 data points for each other’s investigated property at different time points. The data sets are available as Supporting Information.

The kMC-generated data was divided into training and test sets (80 and 20%, respectively) to train and evaluate ML models. Model performance was evaluated for the test set using two common regression metrics: coefficient of determination (*R*^2^) and mean absolute error (MAE).^60,61^*R*^2^ is a scaled metric that reflects how well the model captures the overall trend. This metric indicates the proportion of variance in the target variable that is explained by the input variables. A higher *R*^2^ (maximum value is equal to 1) indicates a better fit but does not indicate how big the errors are on average. MAE metric focuses on the magnitude of the errors between predicted and actual values. The errors are expressed in the same units as the data. Thus, the percentage of these errors to the mean value can be assessed. Additionally, 5-fold cross-validation was performed to obtain an average performance measure for each model. As discussed below, one goal is to determine the minimum amount of data required for reliable predictions. Therefore, in experiments with AI models related to the scalability and robustness, the training set was randomly reduced, keeping the test set of the same constant size of 20% of the full training set.

The hyperparameters of the ML models were optimized by using a grid search. For RF models, the number of trees in the forest is equal to 100 and the maximum depth of the tree is equal to 30, except for the branching level prediction, for which the values are 50 and 20, respectively. The values for the bandwidth or smoothing parameters for multivariable KD regression are selected for each variable and model (Table 1). Smoothing parameters scaled the width of the kernel. This means placing a smooth function at the location of each data point and then summarizing the results. We use Gaussian kernels, and the initial values of the smoothing parameters are selected according to the data-driven AIC^43^ method, which is based on the log-likelihood function. Then, to avoid overfitting, these values were corrected with respect to the values of smoothing parameters obtained by the Scott’s rule^45^ as follows. In the case where the suggested values of a parameter were similar by both methods, the AIC suggestion was chosen. If the values of both methods were significantly different, then a small grid search in the range between the proposed values of both methods was performed.

**Table 1:** Values of the Smoothing Parameters of KD Regression for Each ML Model

KD model	*c*_BA,0_/mol·L^–1^	*T/*°C	time/s
*c*_BA_	0.139	2.48	92.65
MMC	0.08	1.2	179.0
MMD	0.13	1.55	230.88
DP_95_	0.15	1.343	300.7
BL	0.17	1.75	230.8

While evaluating the computational demand of RF and KD regression, training and prediction times turned out to be negligible. Both methods completed these tasks in less than a second. However, there was a small difference: RF training was slightly slower than KD training, and conversely, KD prediction was slightly slower than the RF prediction. This difference became more pronounced when predicting the MMD using KD, as it necessitates predictions for multiple intervals. Even in this scenario, the processing time remained around a second.

The most significant computational hurdle for both methods was the hyperparameter tuning. This step involves evaluating numerous parameter combinations and can take several hours. KD tuning, in particular, maybe even more time-consuming, especially for data sets with a high number of input variables. To address the extensive tuning time for KD, some efficiency-enhancing assumptions were made: (1) The same set of parameters was used for KD across all MMD intervals and BL prediction times; (2) Hyperparameter tuning was performed only while training models on the complete training set. These parameters were then applied to models trained on reduced data sets. While these assumptions improved the efficiency, it is important to acknowledge that they might have introduced slight limitations to the accuracy of the predictions. Avoiding these assumptions could probably lead to slightly improved accuracy, however, at the expense of significantly longer tuning times.

The models are evaluated based on three key aspects:

- *Performance*: Which ML model yields the most accurate predictions for each property?
- *Robustness*: How does model performance degrade with decreasing training data set? Which amount of data is necessary for good and reliable predictions?
- *Explainability*: How do the chosen polymerization parameters (temperature and initial monomer concentration) affect the model predictions? Are the results from explainability methods chemically reasonable?

### 4.2 Prediction of Butyl Acrylate Concentration

**Figure 4:**
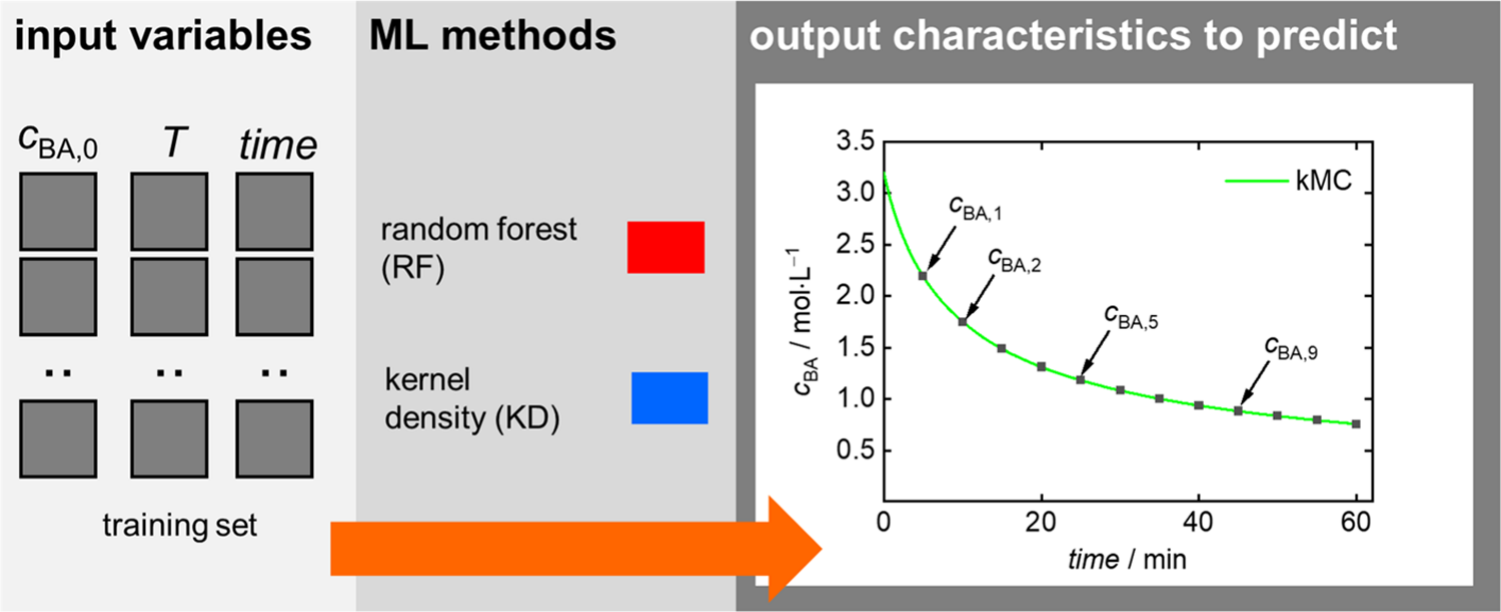
BA concentration prediction as a regression problem. For further details, see the main text.

The RF and the KD regression models use eq 3 with *Y*(*t*) = *c*_BA_(*t*) to predict *c*_BA_ at different time points, as illustrated in Figure 4. The prediction process is optimized by focusing on key points of the *c*_BA_ trajectory. Since the *c*_BA_ profile exhibits a monotonic decrease, it is not necessary to predict every data point. Instead, the crucial turning points and significant moments along the *c*_BA_ curve are identified and approximated. This approach offers a more efficient and target-oriented analysis of the *c*_BA_ behavior.

**Figure 5:**
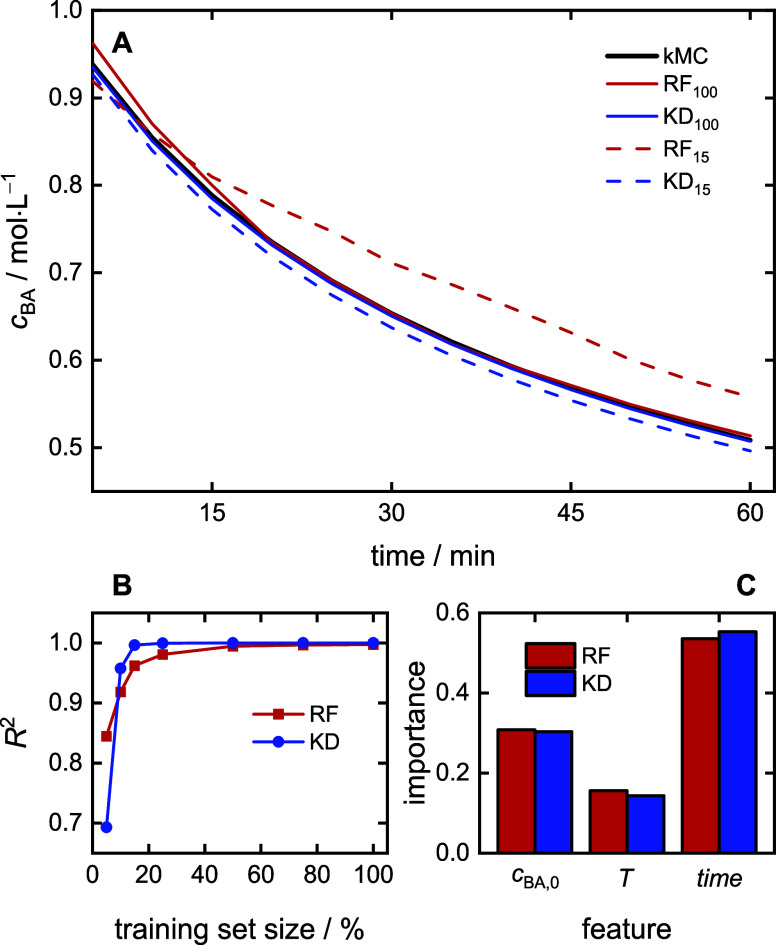
*c*_BA_ prediction models: (A) example for *T* = 134.4 °C, *c*_BA,0_ = 1.05 mol·L^–1^, training data set size: 218 (simulations) × 12 (time points) = 2616, (B) model performance for different training set sizes, and (C) feature importance.

Figure 5 refers to the *c*_BA_ predictions obtained with RF and KD ML models. Figure 5A illustrates a typical prediction example. The models applied are evaluated in the lower parts of Figure 5 and Table 2. The *performance* is assessed on the basis of two metrics, *R*^2^ and MAE. Visual inspection confirms excellent agreement between the predicted *c*_BA_ trajectory from KD regression (blue line) and the kMC-simulated data (black line) for the entire test set, with KD regression achieving the highest *R*^2^ score of 0.999 and RF modeling resulting in a slightly lower *R*^2^ value of 0.997, as indicated in Figure 5B. Table 2 shows that the MAE metric for the whole test set is below 1% of the average *c*_BA_ value for both models, with the error related to the KD approach being 5 times smaller than that for RF. The impact of training set size was investigated to judge the *robustness* of both methods. As expected, performance generally declines with fewer training points for both metrics. However, the KD regression displayed remarkable robustness, maintaining a high *R*^2^ of 0.996 and a MAE of 1% even with only 15% of the training data. While predictions with just 10% of the data suffered from accuracy, which is evidenced by *R*^2^ values of 0.957 and 0.918, as well as by MAE of 3 and 8% for KD and RF, respectively. The advantage of KD’s efficiency remains evident. Predictions with just 5% of the data suffered in accuracy considerably for both models (KD *R*^2^: 0.693, RF *R*^2^: 0.844; KD MAE: 8%, RF MAE: 11%). Analysis of model *explainability* shown in Figure 5C revealed a consistent trend across all models: time is the most significant input variable, contributing around 55% to the prediction. The initial BA concentration (*c*_BA,0_) holds moderate importance (30%), followed by the temperature (15%). From a chemical point of view, the explainability result is reasonable since the monomer concentration decreases steadily with time. On the contrary, the MMD and MMC discussed below are cumulative properties, which depend only slightly on the time. Since the radicals are generated via self-initiation of the monomer, which depends on the monomer concentration and the temperature, considerable importance of *c*_BA,0_ and temperature is understandable as well.

**Table 2:** MAE Performance Metric for *c*_BA_ Prediction Models, the Relative Error Is Calculated for an Average Value of *c*_BA_ Equal to 1.1 mol·L^–1^

	MAE/mol·L^–1^	
training set size/%	RF	KD
100	0.017 (1%)	0.003 (<1%)
75	0.020 (2%)	0.003 (<1%)
50	0.025 (2%)	0.004 (<1%)
25	0.045 (4%)	0.006 (<1%)
15	0.063 (6%)	0.014 (1%)
10	0.086 (8%)	0.033 (3%)
5	0.127 (11%)	0.096 (8%)

### 4.3 Prediction of Macromonomer Content

**Figure 6:**
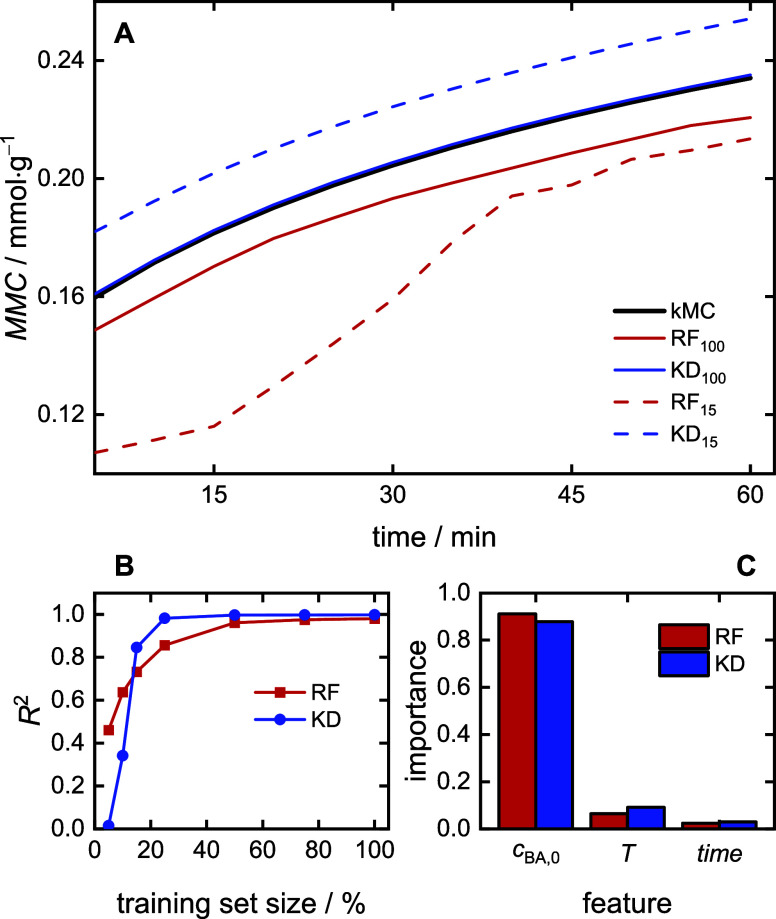
MMC prediction models: (A) example for a prediction with *T* = 135.4 °C, *c*_BA,0_ = 1.35 mol·L^–1^, the training data set size is given, (B) model performance for varying training data set sizes, (C) feature importance.

The regression models use eq 3 with *Y*(*t*) = MMC(*t*) to predict the macromonomer content. Similar to the results presented for *c*_BA_ in Figure 5, KD regression excels in predicting the *MMC* trajectories (Figure 6). As exemplified in Figure 6A, using the full training data set, the prediction based on KD regression perfectly overlaps with the kMC simulation data, while RF regression leads to data that deviates significantly. *Performance* of both models is characterized by *R*^2^ scores higher than 0.98, with the *R*^2^ of the KD prediction reaching 0.998. Table 3 shows that the MAE metric is less than 1% for KD and five times higher for RF than for KD. As expected, performance declines with smaller training sets. However, the KD demonstrates superior *robustness*. Even with only 25% of the training data, KD maintains a strong *R*^2^ of 0.982 and less than 4% for MAE, while *R*^2^ drops to 0.856 and MAE drops to 16% for RF. The result suggests that KD models require significantly fewer training data for accurate MMC prediction. Further reduction of the training set leads to inaccurate predictions.

**Table 3:** MAE Performance Metric for Two Prediction Models^a^

	MAE of MMC/mmol·g^–1^		MAE of MMD	
training set size/%	RF	KD	RF	KD
100	0.006 (6%)	0.001 (<1%)	0.010 (5%)	0.002 (<1%)
75	0.007 (7%)	0.001 (<1%)	0.011 (5%)	0.003 (1%)
50	0.009 (8%)	0.001 (1%)	0.015 (7%)	0.003 (1%)
25	0.016 (16%)	0.004 (4%)	0.023 (11%)	0.006 (3%)
15	0.021 (21%)	0.010 (10%)	0.030 (14%)	0.010 (5%)
10	0.026 (25%)	0.033 (32%)		

a (1) MMC, with the relative error calculated for an average MMC value of 0.1 mmol·g^–1^; (2) MMD, with the relative error calculated for an average value of *w*(log(*M*)) equal to 0.213.

The *explainability* is addressed in Figure 6C. The feature importance analysis reveals a consistent trend across models: initial BA concentration (*c*_BA,0_) is the most influential factor (90%), followed by temperature (8%) and time (2%). This aligns with our understanding of high-temperature acrylate polymerization. As indicated in Figure 1, macromonomers are originating from the β scission reaction of midchain radicals, which is competing with addition of monomer molecules. Therefore, lowering of *c*_BA,0_ reduces the probability of chain propagation of MCRs and favors competing β-scission reactions, leading to higher macromonomer content. It goes without saying that a higher *c*_BA,0_ has the opposite impact on MMC.

### 4.4 MMD Prediction

**Figure 7:**
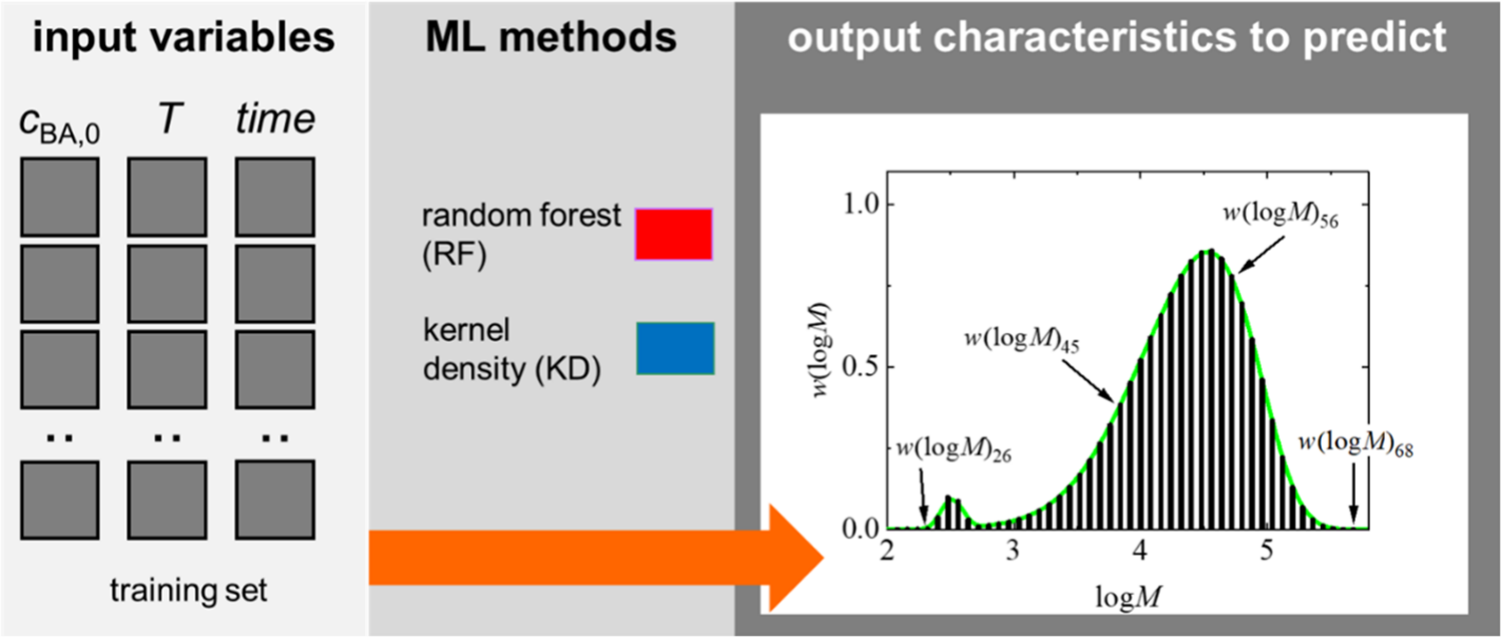
MMD prediction principles with MTR. Further details are provided in the main text.

**Figure 8:**
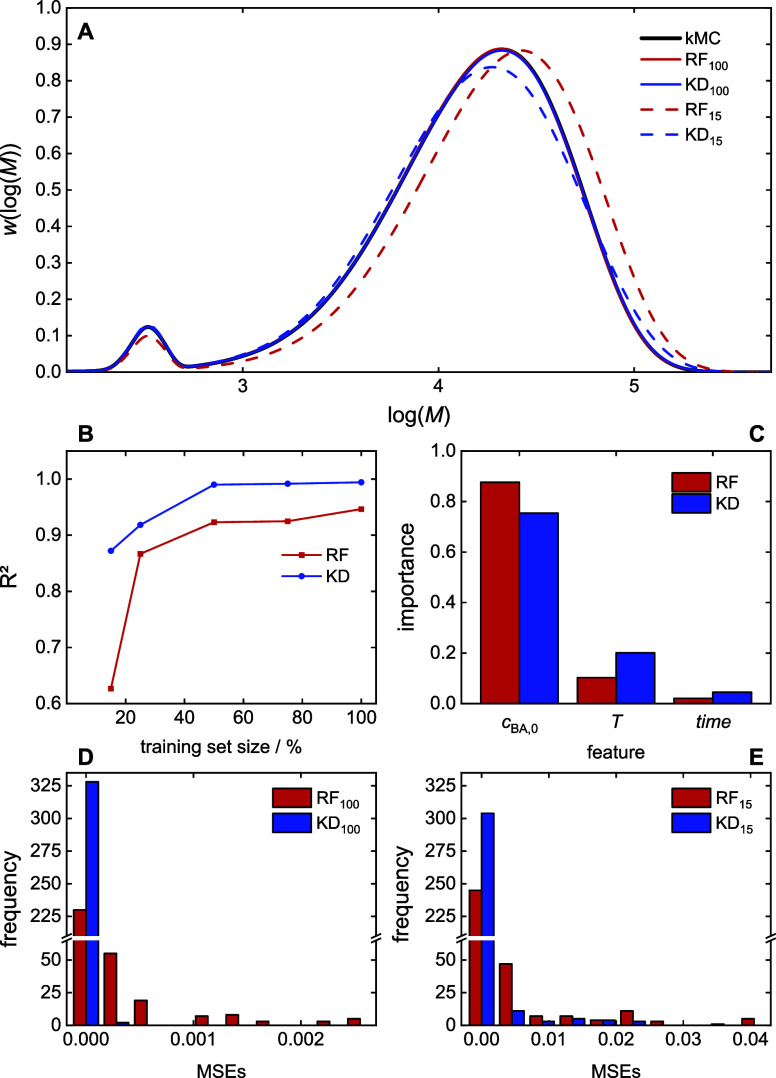
MMD prediction models: (A) example of a prediction for *T* = 137.5 °C, *c*_BA,0_ = 2.767 mol·L^–1^, time = 60 min for the RF and KD methods, (B) model performance, (C) feature importance, (D) MSEs of test set data of the models trained on the full training data sets, (E) MSEs of test set data of the models trained on reduced training data sets.

The prediction of MMDs is a unique challenge because MMDs are distributions represented by vectors contrary to the scalar properties considered so far. To achieve realistic forecasts, we describe MMDs using 100 equidistant grid points, each representing a weight fraction (*w*(log(*M*))) within the distribution, which is illustrated in Figure 7. Given the inherent relation between these weight fractions, a multitarget regression (MTR) model^28,62^ is well-suited for this task. In this model, each output variable corresponds to a specific *w*(log(*M*))_*j*_ fraction interval, with *j* referring to a weight fraction (*w*(log(*M*))). The regression models use functional dependence (3) with *Y*(*t*) = MMD(*t*). To assess the accuracy of MMD predictions, the mean squared error (MSE) was employed as a similarity metric. MSE calculates the average squared difference between corresponding points in two predicted MMDs for a given experiment. Figure 8A showcases an example of MMD predicted by RF and KD models for a single experiment. The corresponding MSE values for each MMD are displayed in Figure 8D,E.

The RF model achieves an average *performance* exceeding *R*^2^ = 0.946. The KD model demonstrates superior performance, with an average *R*^2^ of 0.994. Table 3 shows small values for the MAE metric with less than 1% error for KD and 5% error for RF. As expected, the performance of both models decreases with a reduction in the training data set size, with the KD model exhibiting greater resilience. With respect to *robustness*, it is important to note that even with only 25% of the training data, the KD model maintains an *R*^2^ of 0.918 and MAE of 3%, while the RF model drops to *R*^2^ = 0.867 and MAE = 11%. Further reduction in training data size leads to both models falling below an *R*^2^ of 0.9, indicating insufficient accuracy with MAE values of 14 and 5% for RF and KD models, respectively. This finding suggests that the KD model can provide reliable predictions even with a 75% reduction in training data in contrast to the RF model.

Figure 8C delves into the *explainability* of the models, revealing the impact of different factors on the predicted MMDs. For both models, the initial monomer concentration (*c*_BA,0_) has the most significant influence, contributing over 80% of the prediction. Temperature follows with a contribution of around 15%, and finally, reaction time contributes to around 5%. The explainability results from the models align well with established chemical knowledge. The molar masses are largely determined by the monomer concentration because propagation and radical generation are affected by *c*_BA_ due to self-initiation. In addition, monomer addition to a radical occurs in competition with other elemental reactions, e.g., intra- and intermolecular chain transfer to polymer, or β scission in the case of midchain radicals. The impact of reaction time is minor since radical polymerizations are chain growth polymerizations. In general, already at small reaction times, high molar masses are obtained that do not change to a large degree with increasing time. Moreover, MMDs are cumulative properties. Temperature has a slightly higher importance than time, which is in line with the fact that temperature significantly affects the generation of radicals.^9^

Figure 8D,E depicts histograms of the mean squared errors (MSEs) for both RF and KD models when trained on 100 and 15% of the data, respectively. As expected, the distribution of MSEs shifts toward higher values as the training data size decreases. Despite this reduction, a significant fraction of the MSEs remains below 0.01 even for the 15% training data scenario. The results indicate that both models can achieve reasonably accurate predictions even with a substantial reduction in training data. The KD model appears to exhibit a tighter distribution of MSEs at lower training data sizes, suggesting potentially better generalizability in such cases.

### 4.5 Prediction of DP_95_

**Figure 9:**
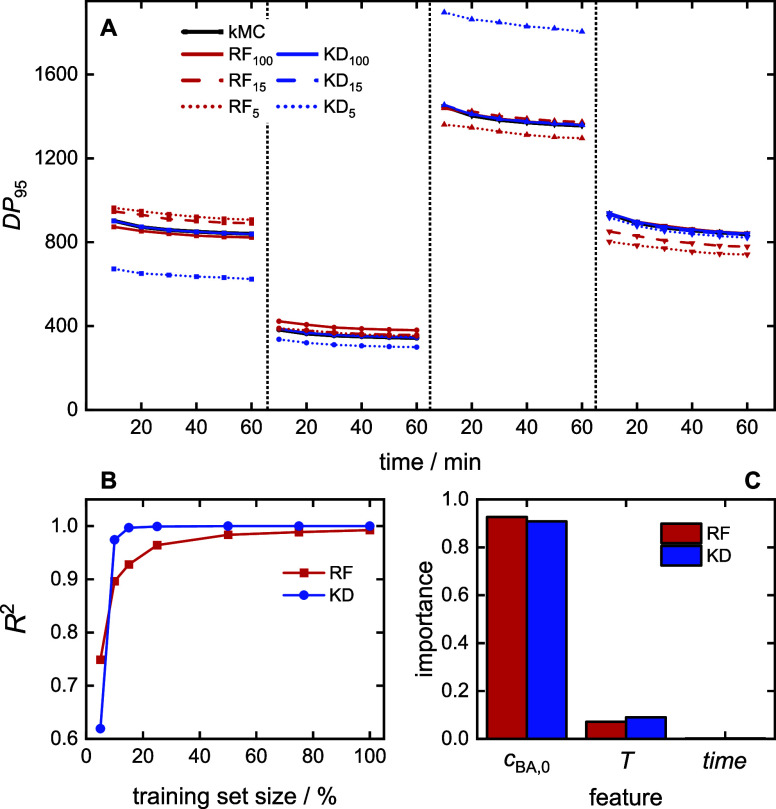
DP_95_ prediction models: (A) four examples for predictions with (1) *c*_BA,0_ = 3.64 mol·L^–1^, *T =* 137.8 °C; (2) *c*_BA,0_ = 2.15 mol·L^–1^, *T =* 136.9 °C; (3) *c*_BA,0_ = 4.26 mol·L^–1^, *T =* 133.7 °C; (4) *c*_BA,0_ = 2.93 mol·L^–1^, *T =* 125.1 °C separated by the dashed lines, (B) model performance, and (C) feature importance.

As discussed in Section 2, the average branching level is closely linked to DP_95_, the cutoff chain length of the MMDs, allowing for reliable branching prediction. Therefore, an ML model for DP_95_ is an important target for prediction. The process of building an ML model for DP_95_ follows similar principles as for the *c*_BA_ model described in Figure 4. The regression models use eq 3 with *Y*(*t*) = DP_95_(*t*). Figure 9A displays the predictions for four example experiments from the test set. Generally, the KD model’s predictions closely align with the data from the kMC simulations, while the RF model exhibits significant deviations.

The *performance* is further assessed by the *R*^2^ presented in Figure 9B and the MAE given in Table 4. When trained with the full data set, all models achieve *R*^2^ values exceeding 0.99, with the KD model reaching an outstanding *R*^2^ of 0.999 and MAE < 1%. The KD model demonstrates greater resilience with respect to decreasing training data. Even with only 25% of the training data, the KD model maintains an *R*^2^ of 0.999 and MAE of 1%, while *R*^2^ is 0.964 and MAE is 9% for RF predictions. It is remarkable that the KD model achieves a very high *R*^2^ of 0.974 and a small MAE of 5% even with only 10% of the training data. A further reduction in the training data results in *R*^2^ below 0.75 and MAE above 22% for both models, indicating insufficient accuracy. The findings demonstrate the *robustness* of the models, with KD predictions being the most reliable.

**Table 4:** Performance Metrics for Two Prediction Models^a^

	MAE of DP_95_		MAE of BL	
training set size/%	RF	KD	RF	KD
100	44 (5%)	5 (<1%)	0.011 (2%)	0.002 (<1%)
75	46 (5%)	5 (<1%)	0.013 (3%)	0.002 (<1%)
50	56 (6%)	6 (1%)	0.016 (3%)	0.002 (<1%)
25	84 (9%)	11 (1%)	0.025 (5%)	0.003 (<1%)
15	119 (13%)	20 (2%)	0.032 (7%)	0.005 (1%)
10	155 (17%)	49 (5%)	0.046 (10%)	0.009 (2%)
5	231 (25%)	201 (22%)	0.072 (15%)	0.047 (10%)

a (1) DP_95_ with the relative error calculated for an average value of DP_95_ equal to 912.5; (2) BL prediction models with the relative error calculated for an average value of BL equal to 0.466.

Figure 9C explores the *explainability* of the models, revealing the factors influencing the predicted DP_95_ values. Consistent with the MMD prediction findings, the initial monomer concentration emerges as the dominant factor for both models, contributing around 90% of the prediction. Temperature follows with a contribution of approximately 8%, and the reaction time has a minor influence of around 2%. The chemical rationale behind these results aligns with the explainability of MMD prediction. Since multiplying chain length with the constant BA molar mass yields the polymer molar mass, factors impacting MMD also significantly affect DP_95_.

### 4.6 Prediction of the Average Branching Level

**Figure 10:**
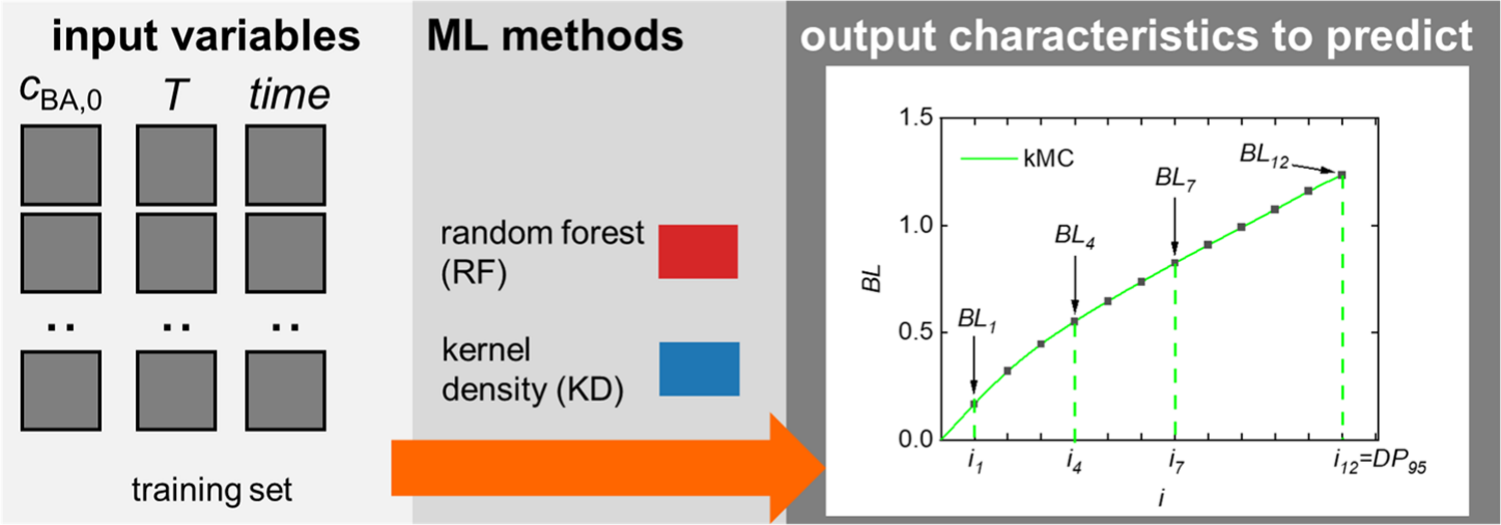
Average branching level prediction principles with multitarget regression. Further details are provided in the main text.

The prediction of an average branching level is more complex as it is a vector similar to MMD. Realistic forecasts involve dividing the chain length distribution into 12 intervals (*i*_1_ – *i*_12_) based on predicted DP_95_. Then, a multitarget regression model is applied, where each output variable corresponds to a BL value for a specific chain length interval BL(*i*) as illustrated in Figure 10. The regression models use eq 3, with *Y*(*t*) = BL(*t*).

**Figure 11:**
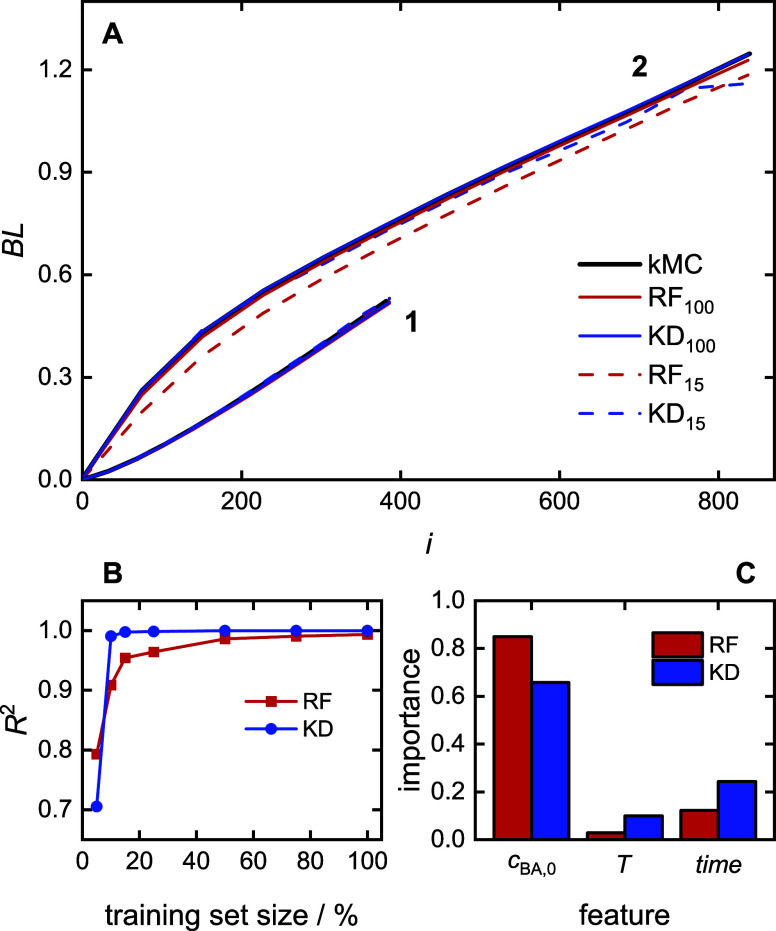
Branching level prediction models: (A) examples of predictions with the RF and the KD method for two different experiments: (1) *T* = 132 °C, *c*_BA,0_ = 2.75 mol·L^–1^, time = 60 min; (2) *T* = 130 °C, *c*_BA,0_ = 1.85 mol·L^–1^, time = 10 min; (B) model performance, (C) feature importance.

Figure 11 provides the prediction results. For the two example experiments given in Figure 11A, predictions of both models closely align with the kMC simulation data. According to Figure 11B and Table 4, the excellent *performance* is reflected by *R*^2^ scores higher than 0.99 and MAE lower than 2%, with *R*^2^ values of 0.999 and MAE values of less than 1% for KD predictions. Even with only 15% of the data, the KD method maintains a strong *R*^2^ > 0.997 and a MAE of 1%, while *R*^2^ for the RF approach drops below 0.96 and provides MAE of 7%. Both results indicate the high *robustness* of the BL predictions. It is very remarkable to note that KD regression is associated with an *R*^2^ of 0.99 and MAE of 2% with only 10% of the training data, which suggests significantly lower training data requirements for KD models. With 5% of the training data, the *R*^2^ performance drops below 0.8 and MAE is 10% for KD, while *R*^2^ = 0.71 and MAE = 15% for RF models. Therefore, a minimum of 10% training data is recommended for BL prediction with KD regression.

As for the previous analyses, the *explainability* results depicted in Figure 11C show consistent trends across models. The initial BA concentration is the dominant factor with a 70% contribution, followed by time and temperature contributing with 20 and 10%, respectively. As discussed in detail for the MMDs, the monomer concentration has the highest impact on the results. With respect to BL, the monomer addition and the chain transfer to polymer reactions must be considered. A change in *c*_BA,0_ significantly alters the probabilities of self-initiation, propagation of midchain radicals, and transfer, thus affecting the occurrence of branch points and consequently the average degree of branching. The small impact of time is in line with the fact that BL is also a cumulative property. The temperature influence is minor because the competition of both reactions mentioned is not significantly affected by temperature.

## 5 Conclusions

This study bridges the gap between cutting-edge machine learning methods and their application in modeling high-temperature polymerization processes. We propose a suite of highly accurate ML models capable of predicting crucial properties like molar mass distributions, average branching level, and macromonomer content with exceptional performance. The KD repression proves to be particularly suited with *R*^2^ values higher than 0.99.

The key achievements are

- **Validated and scalable ML models:** A robust and scalable set of ML models was established that facilitate fast and efficient training using data derived from kMC simulations. These models predict four key properties: variation of monomer concentration, macromonomer content, and molar mass distributions with time and the branching level as a function of the chain length.
- **Data-efficient learning:** The proposed KD regression model consistently outperforms RF model, achieving remarkable performance (*R*^2^ > 0.99 and MAE < 1%) even with reduced training data. This significantly reduces the computational demand of kMC simulations and potentially allows for training using real-world lab data.
- **Transparent and chemically meaningful models**: Explainability methods revealed the importance of different input variables, aligning well with polymer engineering principles. This transparency fosters trust in the models’ predictions.

The proposed models hold immense potential for future research, particularly in the reverse engineering of polymerizations. By utilizing a genetic algorithm-based multiobjective optimization approach, these models can be instrumental in determining the ideal recipe for synthesizing polymers with desired properties. The BL prediction, along with MMD, conversion, and time, can serve as key optimization objectives.

## Supporting Information Available

Provided are monomer concentration and macromonomer content as a function of time, molar mass distributions, and branching level evolution throughout the polymerization (XLSX)
